# 2501. Trends in viral hepatitis and hepatocellular carcinoma in New York City, 2001–2018

**DOI:** 10.1093/ofid/ofad500.2119

**Published:** 2023-11-27

**Authors:** Ned H Latham, Holly Anger, Angelica Bocour, Baozhen Qiao, Tabassum Insaf, Tristan D McPherson

**Affiliations:** New York City Department of Health and Mental Hygiene, Sydney, New South Wales, Australia; New York City Department of Health and Mental Hygiene, Sydney, New South Wales, Australia; New York City Department of Health and Mental Hygiene, Sydney, New South Wales, Australia; New York State Department of Health, Albany, New York; New York State Department of Health, Albany, New York; New York City Department of Health and Mental Hygiene, Sydney, New South Wales, Australia

## Abstract

**Background:**

In New York City (NYC), an estimated 243,000 people live with chronic hepatitis B virus (HBV) infection and 86,000 people live with chronic hepatitis C virus (HCV) infection, representing 2.9% and 1% of the population, respectively. HBV and HCV can cause liver fibrosis, cirrhosis, and hepatocellular carcinoma (HCC). Early diagnosis improves HCC survival, and regular screening of people with HBV and other risk factors (e.g., family history of HCC or active liver inflammation) or HCV with cirrhosis is recommended. We aimed to describe recent trends in viral hepatitis and HCC in NYC.

**Methods:**

We generated a retrospective cohort of NYC residents with HCC by matching New York State Cancer Registry data with NYC Health Department viral hepatitis surveillance data. We included all HCC cases diagnosed from 2001-2018 in the analyses, except those reported solely by Veterans Affairs hospitals or other state Cancer Registries. We defined people as having HBV and/or HCV if they met the Council for State and Territorial Epidemiologists’ probable or confirmed case definition before, or within 30 days of, their HCC diagnosis. We analyzed annual incidence of HCC stratified by viral hepatitis status (HBV, HCV, coinfection, neither), and the proportion of patients diagnosed with HCC at the local stage (vs. regional or distant stage).

**Results:**

Between 2001–2018, 13,209 NYC residents were diagnosed with HCC; 2,456 (18.6%) had HBV, 5,379 (40.7%) had HCV, and 282 (2.1%) had HBV/HCV coinfection (Table 1). Incident HCC cases increased from 495 in 2001 to a peak of 878 cases in 2012 (Figure 1). The number of HCC cases with HBV or no viral hepatitis was relatively stable over the period, while HCC cases with HCV increased steadily until 2012, then declined slightly from 2016 to 2018. The proportion of HCC cases diagnosed at the local stage generally increased for people with HBV (48.3 to 60.7%), HCV (52.6% to 59.3%), and neither infection (41.4 to 57.6%).

**Figure 1.**
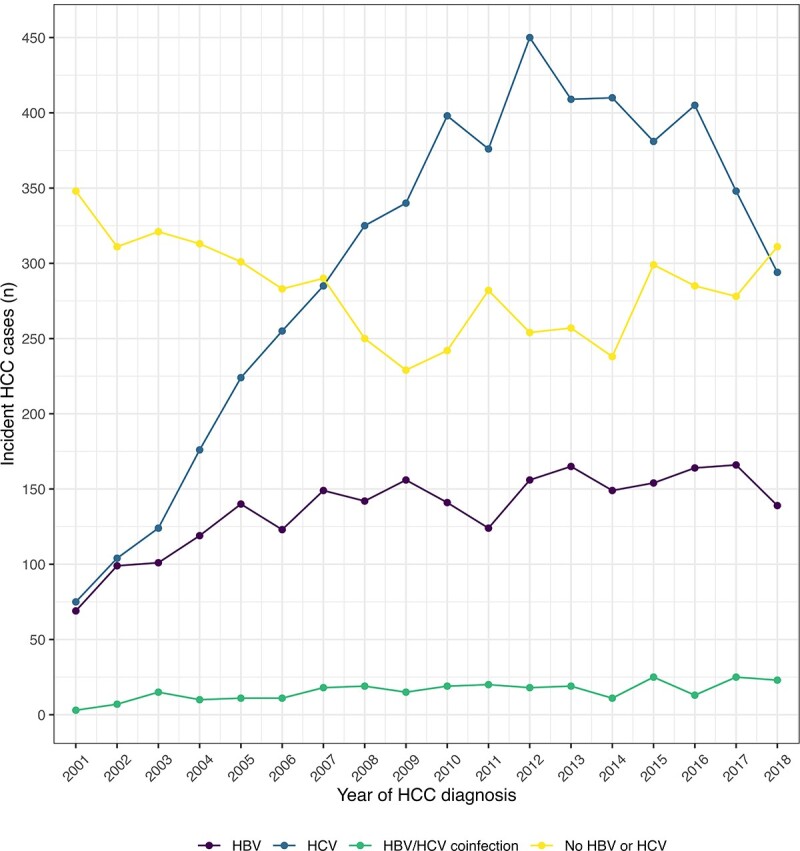
Trends in viral hepatitis status of incident hepatocellular carcinoma (HCC) cases among New York City residents, 2001–2018. HBV = hepatitis B virus HCV = hepatitis C virus HCC = hepatocellular carcinoma

**Table 1.**
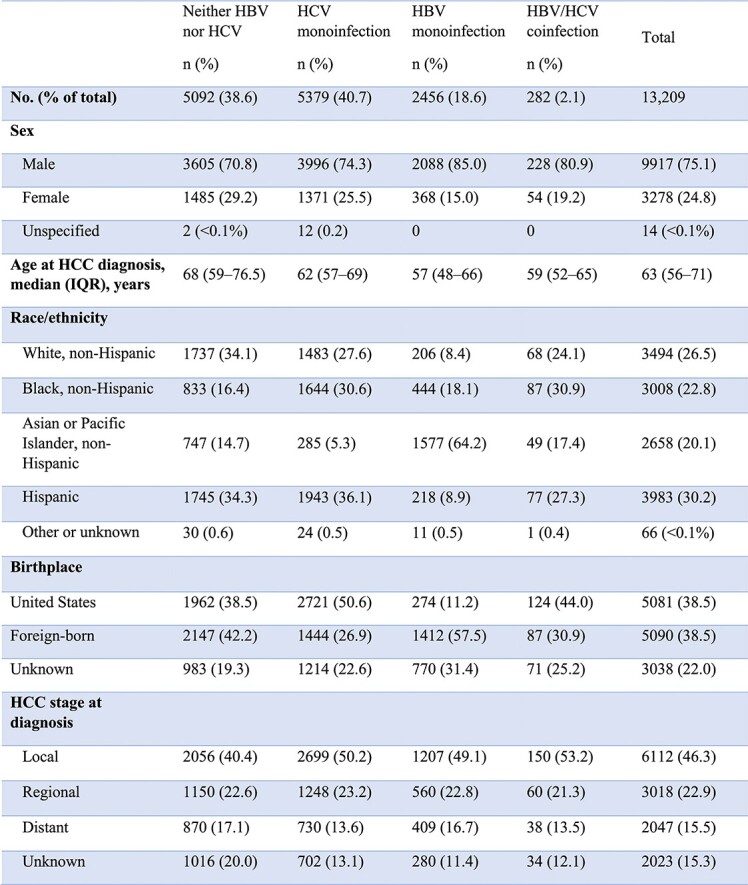
Characteristics of people diagnosed with hepatocellular carcinoma with and without viral hepatitis in New York City, 2001–2018.

IQR = interquartile range HCC = hepatocellular carcinoma Due to rounding, category percentages do not all sum to 100.

**Conclusion:**

Recent declines in HCC among NYC residents reflect declines in HCC cases with HCV. Despite increases in the proportion of HCC cases diagnosed at the local stage, opportunities to improve screening remain. Additional analyses accounting for antiviral treatment, cirrhosis, alcohol intake, and mortality, would assist in further informing HCC prevention efforts.

**Disclosures:**

**All Authors**: No reported disclosures

